# Shifting Effects of Ocean Conditions on Survival and Breeding Probability of a Long-Lived Seabird

**DOI:** 10.1371/journal.pone.0132372

**Published:** 2015-07-13

**Authors:** Annie E. Schmidt, Kristen E. Dybala, Louis W. Botsford, John M. Eadie, Russell W. Bradley, Jaime Jahncke

**Affiliations:** 1 Department of Wildlife, Fish, and Conservation Biology, University of California Davis, Davis, California, United States of America; 2 Point Blue Conservation Science, Petaluma, California, United States of America; Norwegian Polar Institute, NORWAY

## Abstract

With a rapidly changing climate, there is an increasing need to predict how species will respond to changes in the physical environment. One approach is to use historic data to estimate the past influence of environmental variation on important demographic parameters and then use these relationships to project the abundance of a population or species under future climate scenarios. However, as novel climate conditions emerge, novel species responses may also appear. In some systems, environmental conditions beyond the range of those observed during the course of most long-term ecological studies are already evident. Yet little attention has been given to how these novel conditions may be influencing previously established environment–species relationships. Here, we model the relationships between ocean conditions and the demography of a long-lived seabird, Brandt’s cormorant (*Phalacrocorax penicillatusI*), in central California and show that these relationships have changed in recent years. Beginning in 2007/2008, the response of Brandt’s cormorant, an upper trophic level predator, to ocean conditions shifted, resulting in lower than predicted survival and breeding probability. Survival was generally less variable than breeding probability and was initially best predicted by the basin-scale forcing of the El Niño Southern Oscillation rather than local ocean conditions. The shifting response of Brandt’s cormorant to ocean conditions may be just a proximate indication of altered dynamics in the food web and that important forage fish are not responding to the physical ocean environment as expected. These changing relationships have important implications for our ability to project the effects of future climate change for species and communities.

## Introduction

Ecologists have long been interested in the effects of environmental variability on populations. More recently, the threat of global climate change has made efforts to understand these effects increasingly urgent. A useful approach is to use demographic time series to establish how vital rates (i.e. survival and reproduction) varied in response to key environmental variables in the past, and then use these empirical relationships to project a population’s response to predicted future climate (e.g. [1,2]). However, the ecological data on which environment-species relationships are based rarely span more than a few decades and have been collected under a limited range of climate conditions. As novel climates emerge, species ranges may shift, no-analog communities may develop [3], and some of these empirical environment-species relationships may be disrupted, resulting in novel species responses and ecological surprises [3,4]. Relationships that change unexpectedly could lead to erroneous or misleading population projections.

In the Pacific Ocean, conditions outside the range of observations of the past few decades are already apparent. For example, variance is increasing in an important large-scale forcing pattern, the North Pacific Gyre Oscillation (NPGO)[5], the spatial distributions of El Niño events are shifting [6–8], and the intensity of wind driving coastal upwelling is increasing [9,10]. These are just a few of the recently observed changes that may have already produced deviations in established environment-species relationships. In a previous analysis, we found evidence to suggest that the reproductive success of seabirds in central California is responding differently to ocean conditions than it has in the past and that the effects differ for two species feeding at different trophic levels [11]. In particular, we showed that the relationships between ocean conditions and the reproductive success of an upper trophic level predator, Brandt’s cormorant (*Phalacrocorax penicillatus*) changed such that the relationships that had been established for nearly 30 years disappeared beginning between 2007 and 2008 [11]. The ocean conditions in 2007/2008, although cool, were not particularly unusual and were within the range of conditions that had occurred before in the time series. What was noteworthy was the unusual response of the seabirds. Specifically, cool ocean conditions that are typically associated with high food web productivity resulted in near complete reproductive failure for the piscivorous Brandt’s cormorant [11].

Although the exact mechanism driving the shifting relationship remains uncertain (but see [11] for discussion of possible mechanisms), it is important to understand the consequences these shifts may have for long-term population dynamics, a problem that requires first understanding how these changes may impact multiple vital rates. In our previous analysis, we documented a change in the response of just one vital rate, reproductive success. However, there are several additional parameters that contribute importantly to population dynamics. For example, in long-lived species the population growth rate tends to be more sensitive to variation in survival than reproduction [12,13]. Thus, changes in the environment-survival relationship may be more important than changes in the environment-reproduction relationship. Further, for long-lived species in highly stochastic environments, individuals faced with the trade-off between reproducing under harsh conditions and the potential cost of lowered survival, may skip breeding altogether [14–16]. This strategy allows species to buffer survival by adjusting reproductive output but can result in higher variability in reproductive parameters. Additionally, survival and breeding parameters can exhibit disparate even opposite responses to the same environmental conditions (as well as depend on the age and sex of individuals, e.g. [17,18]). For example, the survival of Eurasian oystercatcher (*Haematopus ostralegus*) increased with warmer winter temperatures while at the same time, fecundity decreased [19]. Hence, the population growth rate may be most sensitive to variation in adult survival but breeding probability may respond differently than survival to environmental variation and contribute significantly to population fluctuations [12,20]. Although variation in survival with respect to environmental conditions has been previously documented in this Brandt’s cormorant population [17], the effect of environmental conditions on breeding probability has not been examined.

Given the potential importance of changes in adult survival and breeding probability to a population, here we follow up on our previous analysis of reproductive success of Brandt’s cormorant to examine the impact of shifting ocean conditions on these additional demographic parameters. Specifically we ask: 1) are relationships between ocean conditions and survival and breeding probability of Brandt’s cormorant stable (stationary) or did these relationships also change around 2007/2008? 2) do survival and breeding probability respond in the same way to ocean conditions? The stability of these types of relationships is rarely examined, due in part to the relative scarcity of long-term ecological and demographic data. We use over 40 years of data to provide an unusually robust historical context in which to place recent changes.

## Materials and Methods

### Field Methods

Point Blue Conservation Science (formerly Point Reyes Bird Observatory) has monitored the breeding activity of Brandt’s cormorant (S1 Fig) on Southeast Farallon Island continuously since 1970. Monitoring was non-invasive and conducted from an observation blind overlooking the main study colony. No adults were handled and all marked individuals were banded as chicks (all individuals in the study are known-age). Chicks were banded just prior to fledging and received an individually coded metal band and single color band in a combination specific to their cohort. Beginning in 2002, the color band included an engraved 3 digit alphanumeric or alpha code. There were several years when no bands were deployed, due primarily to poor reproductive success and no chicks reaching banding age (S2 Fig). Marked individuals were resighted by reading metal and/or color band numbers from the observation blind (a distance of ~20-100m from nests) using a 20-60x power spotting scope. To minimize “false-positives” due to band reading errors, we only considered band numbers that had been recorded at least twice in a resighting period to be present during that year (similar to [17]). Resighting occurred throughout the breeding season (April-August) each year and we estimated annual survival from the start of one breeding season to the next (April-March). All fieldwork on the Farallon National Wildlife Refuge was conducted under co-operative agreement 81640AJ008 between Point Blue Conservation Science and the US Fish and Wildlife Service. Cormorant banding was conducted under Point Blue’s Federal Banding Permit 09316 from the Bird Banding Laboratory, US Department of Interior.

### Mark-recapture analysis

**Fig 1:**
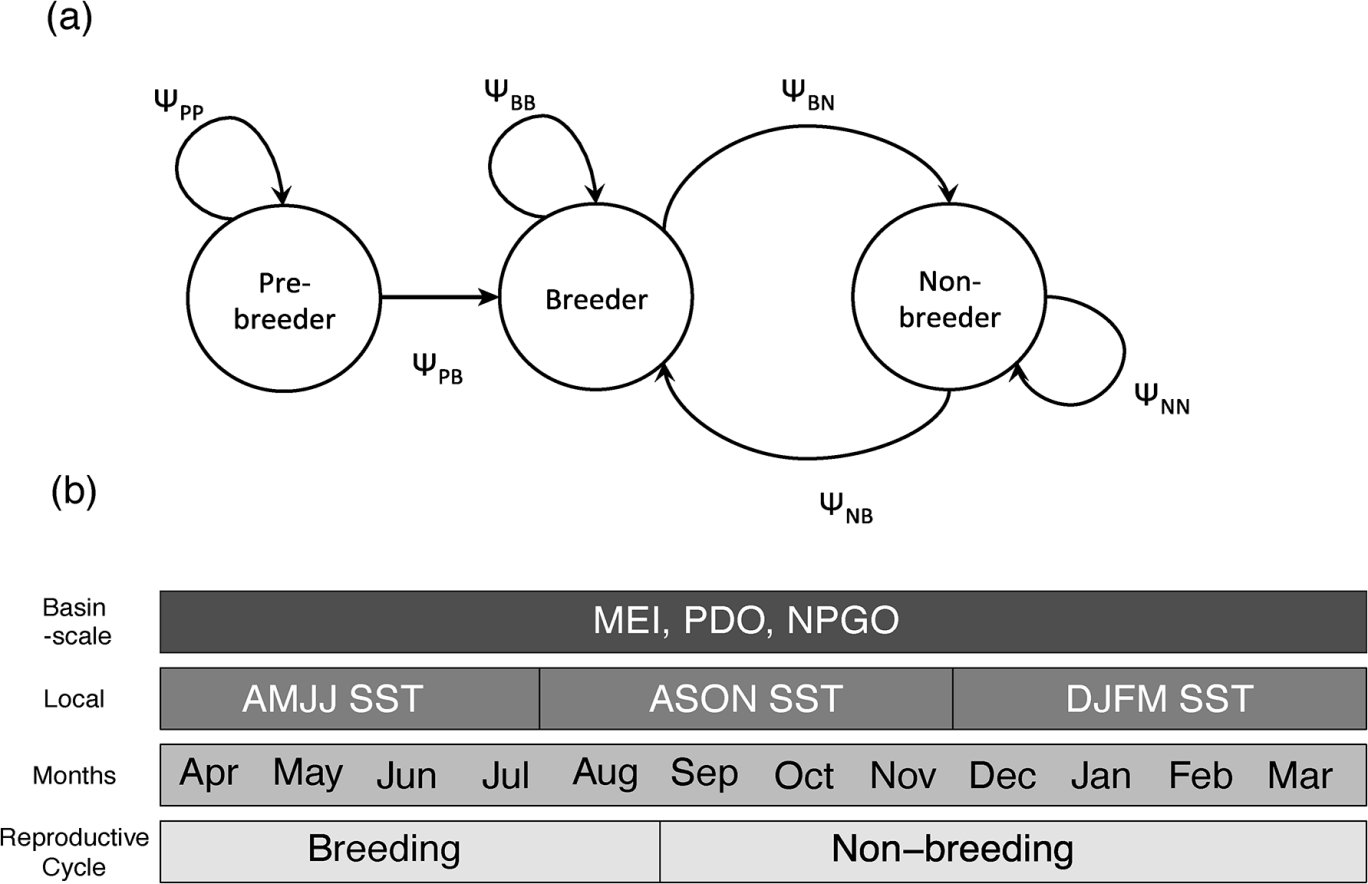
State transitions and timing of ocean covariates. (A) Diagram of the possible breeding state transitions for Brandt’s cormorant. In each state, an individual can either stay or transition to one other state each year. The transitions Ψ_PB,_ Ψ_BB_, and Ψ_NB_ (arrows pointing to the Breeder state) are estimated in the models. All other transitions (complements) are computed by subtraction. (B) Breeding season and period that each ocean covariate was averaged over. Recaptures occurred throughout the breeding season.

We used multistate mark-recapture models run in program MARK (compiled for linux) using the R package RMark [21–24]. We chose multistate models so that we could model the probability of transitioning between breeding states in addition to modeling annual survival and recapture probabilities. Most Brandt’s cormorants do not return to the colony until at least age 2 when some individuals begin breeding. A small fraction (<5%) return to the colony and are observable at age 1 but do not breed [25]. We classified individuals into three states based on breeding status [26]: pre-breeder (individuals that have never been recorded breeding), breeder (individuals confirmed breeding in a given year), and non-breeder (bred at least once previously, observed at the colony not breeding). Because all individuals were initially marked as chicks, all birds in our study started in the pre-breeding state and could not transition into the breeding state until at least age 2 (pre-breeder to breeder transition probability constrained to be 0 until age 2). We added several additional constraints to the model such that individuals in the pre-breeding state could only transition into the breeding state and individuals in the breeding and non-breeding states could not transition back to become pre-breeders (Fig 1A). Once in the breeding state, individuals could either stay in that state or transition to non-breeder each year. Non-breeders could stay as non-breeders or transition back and become breeders again (Fig 1A). Breeding probability was estimated as three separate transition probabilities: 1) pre-breeder to breeder, 2) breeder to breeder, and 3) non-breeder to breeder.

### Goodness-of-fit

Testing the Goodness-of-fit for multistate models is limited to the general Jolly-Move Model (JMV). In the JMV model, transitions depend on the state of departure as well as the state and time of arrival. There is no age dependence included. We performed a goodness-of-fit procedure on the JMV model for our data using program U-CARE to examine potential sources of overdispersion [27]. The data failed both test 3G (transience) and the M-test (trap-dependence) but most of the overdispersion could be attributed to the presence of transients in the pre-breeding state (S1 Table). The detection of transients in the pre-breeding state was likely caused by the extremely low return rates of the youngest age class, an effect that can be modeled by adding age structure to the models [27]. Because the JMV model was not equivalent to our global model, we used the median $\hat{c}$ procedure (100 simulations with a custom routine in R using RMark) to estimate the overdispersion parameter ($\hat{c})$ for the global model (described below). The estimate of $\hat{c}$ obtained confirmed that overdispersion was low after including age structure (estimated $\hat{c}$ = 1.49). Consequently, we included age structure in each parameter of the model. We adjusted model variances using the median $\hat{c}$ estimate and used quasi-Akaike Information Criterion corrected for small sample sizes (QAICc) in all model selection procedures [28].

### Structural Reference Model Selection

We followed the approach of Grosbois et al. [29] and first modeled structural variation using fixed effects of age and year to identify a parsimonious reference model (*Ref*_*t*_) that described the temporal variability in survival, breeding probability and recapture/resighting probability. Once the reference model (*Ref*_*t*_) was identified, we compared it to models where variation in survival or breeding probability was related to environmental covariates. We began with a general (global) model that included additive effects of age, breeding state, and year for all three parameters: apparent survival (S), transition (breeding) probabilities (Ψ), and recapture probability (*p*):

$$Global\;model=\;S_{\left(age+state+year\right)}\Psi_{\left(age+state+year\right)}p_{\left(age+state+year\right)}$$ (1)

Time (year) could only be modeled as an additive effect because the data were too sparse in some years to support a full time interaction with either breeding state or age. Age was modeled in the global model as individual year categories from age 0–11. Individuals age 12 and older were pooled because of small sample size.

We carried out structural model selection by first holding S and Ψ in their most general form (i.e. including effects of age, breeding state and year) and reducing the form of *p*. We reduced the form of *p* by first testing several methods of modeling the age effect. We considered two methods of categorizing age as well as four continuous trends with age. The first age class structure contained two groups, immature (ages 0–1), and adult (ages 2+), since age 2 is the earliest an individual can recruit to the breeding population. We also considered age classification with four classes that corresponded to immature (age 0–1), early breeders (age 2–4), mature breeders (age 5–11) and old individuals (age 12+). The effect of age on demographic parameters may also change continuously with time. For example, survival may increase continuously as individuals gain experience (e.g. linear function), it may plateau at middle age before declining due to senescence (e.g. quadratic function), may initially increase rapidly as young gain experience then slow into adulthood and old age (e.g. log or inverse function of age). Accordingly, we tested four continuous functions: a linear and quadratic effect of age, log (age+1), and an inverse function of age (1/age). Once the most parsimonious age structure was selected, we then examined simpler structures of *p* by removing effects of year and/or state.

When the structure of *p* was reduced to its most parsimonious form we proceeded with the same strategy on Ψ while holding *p* in its most parsimonious and S in its most general form. Finally, we reduced S while holding *p* and Ψ in their most parsimonious forms. We also tested for a trend in survival over time by adding year as a continuous variable with a linear or quadratic trend. A specific model structure was considered supported if the ΔQAICc of the top model and the next best competing model was greater than two [28].

### Relationships with ocean covariates

We chose four variables to quantify ocean conditions, including three basin-scale and one local-scale measure (information on oceanographic data sources contained in S2 Table). The local variable was sea surface temperature (SST), measured daily at the breeding colony (see [30] for full details on collection method). SST is related to the amount of upwelling near the colony with stronger upwelling leading to lower sea surface temperature [31]. Upwelling brings nutrients to the surface and is one of the primary physical processes determining annual productivity in the California Current [31]. The El Niño Southern Oscillation (ENSO), alternates between warm El Niño periods and cool La Niña conditions. During El Niño events, upwelling is reduced, SST typically increases, productivity of the California Current declines, and large-scale reproductive failures of seabirds have been observed [11,31,32]. The opposite occurs during La Niña conditions with increased upwelling, cool SST, and generally an increase in overall productivity in the California Current (e.g. [33,34,35]). The climatic definition of an El Niño/La Niña event refers to a specific SST threshold that is met for a period of months [36]. Rather than categorize years as El Niño or La Niña events, we used the multivariate ENSO Index (MEI) to characterize basin-scale El Niño conditions on a continuous scale. The MEI fluctuates on an annual temporal scale and indicates variation in ENSO as it is expressed at the equator. Positive values of MEI are associated with warmer conditions in the tropical Pacific and negative values of the MEI are associated with cooler conditions [37].

In addition to SST and MEI, we considered two variables describing basin-scale, low-frequency variation in the North Pacific: the Pacific Decadal Oscillation (PDO) and the North Pacific Gyre Oscillation (NPGO). The PDO describes the dominant mode of spatial variability in sea surface temperature in the North Pacific (poleward of 20°N). Its temporal signature is an El Niño-like pattern that varies on a decadal rather than the annual temporal scale and it has been shown to be important in biological productivity [38]. In contrast, the NPGO index is defined as the second dominant mode of spatial variability in sea surface height in the Northeast Pacific. It is associated with decadal scale variations in the circulation of the North Pacific Gyre and correlates well with salinity and several measures of biological productivity in the California Current [39–41].

Survival was estimated from April (year t) to March of the following year (t +1), so for each low-frequency, basin-scale variable, we calculated the mean over 12 months beginning in April at the start of the resight period/breeding season, through March just prior to the next breeding season. To detect a possible seasonal response of survival and breeding probability to conditions near the colony, we calculated seasonal means for the local variable SST. The seasons generally correspond to the spring upwelling season (Apr-Jul), the low upwelling fall season (Aug-Nov), and the winter storm season (Dec-Mar; Fig 1B)[42,43]. All covariates were then standardized to have a mean of 0 and standard deviation of 1. Although some of these variables are correlated (S3 Table) we viewed them each as representing important, though not entirely distinct, processes. For example, the basin-scale processes measured by the MEI also influence local SST. However, there are additional local processes (e.g. strength and duration of northwest wind) that may contribute to variability in local SST, which in turn may have important consequences for local prey availability. Therefore, we wanted to examine the extent to which variables at both the local and basin scale could account for variation in cormorant survival and breeding probability.

We examined the effect of each of these environmental variables on survival and breeding probability by fitting models where the fixed effect of year in *Ref*_*t*_ was replaced with one of four possible continuous relationships with each covariate. The four models tested the shape of the relationship (linear or quadratic) as well as the stability of the relationship. A quadratic relationship may be possible if there is some optimum in oceanographic conditions above and below which cormorants respond less favorably. For example, too much wind and upwelling can advect nutrients and plankton offshore, preventing necessary prey aggregations from forming. On the other hand, too little upwelling may result in reduced availability of nutrients and less biological productivity overall [44]. The stability of the linear or quadratic trends was assessed by adding an interaction with an indicator variable that allowed the model to fit a different intercept and slope for the environmental covariate before versus after 2007 (indicator variable hereafter referred to as After07). The After07 factor was a binary variable coded as zero up to 2006/2007 and one for 2007/2008 and later. Using QAICc, we first selected which relationship (i.e. linear or quadratic, with or without an interaction with After07 factor) was favored for describing the relationship between each covariate and either survival or breeding probability. We then compared each top covariate model with *Ref*_*t*_ and a time constant model (*Ref*_*cst*_). For both survival and breeding probability, QAICc heavily favored the *Ref*_*t*_ model over any covariate model indicating high residual unexplained variance in all covariate models. In this situation, model selection by an information theoretic approach may not be the most efficient for detecting important climatic effects [29]. Accordingly, we performed an analysis of deviance (ANODEV) on the top models for each covariate to assess their fit relative to the fit of *Ref*_*cst*_ and *Ref*_*t*_. We calculated the F test statistic following Skalski [45] and Grobois et al. [29]:

$$F_{k-1,\omega -K}=\;\frac{\left(Dev\;\left(Ref_{cst}\right)-Dev\left(Cov\right)\right)/\left(K-1\right)}{\left(Dev\left(Cov\right)-Dev\left(Ref_{t}\right)\right)/\left(\omega -K\right)}$$ (2)

Where *ω* is equal to the number of survival estimates obtained from the *Ref*_*t*_ model and *K* is the number of parameters required to describe the relationship between survival and the climate covariate [29]. We also assessed how well each covariate accounted for the estimated temporal variability in survival or breeding probability by calculating the R^2^ deviance (R^2^ Dev). The R^2^ Dev for a covariate model is the difference between the model deviance of *Ref*_*cst*_ and the deviance of the model including the ocean covariate (*cov*), proportional to the difference between the deviance of *Ref*_*cst*_ and the *Ref*_*t*_ [29,45].

$$R^{2}Dev=\;\frac{Dev\left(Ref_{cst}\right)-Dev\left(Cov\right)}{Dev\left(Ref_{cst}\right)-Dev\left(Ref_{t}\right)}$$ (3)

### Changing Relationships

In addition to testing models with a change at 2007/2008 (interaction with After07 indicator variable), we also investigated potential shifts in the relationships at different times by performing a sliding correlation analysis of ocean covariates and survival and breeding probability estimates from *Ref*_*t*_. Consistent with our previous analysis [11], we used a 10-year sliding window to calculate the Pearson correlation coefficient between demographic parameters and each oceanographic variable. Because we did not incorporate the uncertainty of the survival and breeding probability estimates into the correlation calculation, we did not calculate *P*-values for the correlation coefficients and view these simply as a qualitative measure to establish whether relationships might have shifted at a point other than 2007/2008.

## Results

From 1970–2012, 12,691 individuals were banded at the study colony (S2 Fig) and 8882 resights were recorded, representing 1111 individuals (8.7% total bands deployed) that were resighted more than once in at least one season. We excluded 1510 individual resight records (17% of 8882 total resights) representing band numbers that were seen only once in a given season. Some of these records are likely legitimate resights but represent transient individuals that only visited the colony briefly and were either not breeding or breeding outside the study area. Removing them may have resulted in estimates of immature survival and recapture probability and non-breeding recapture probability that are biased low.

### Reference model

**Fig 2:**
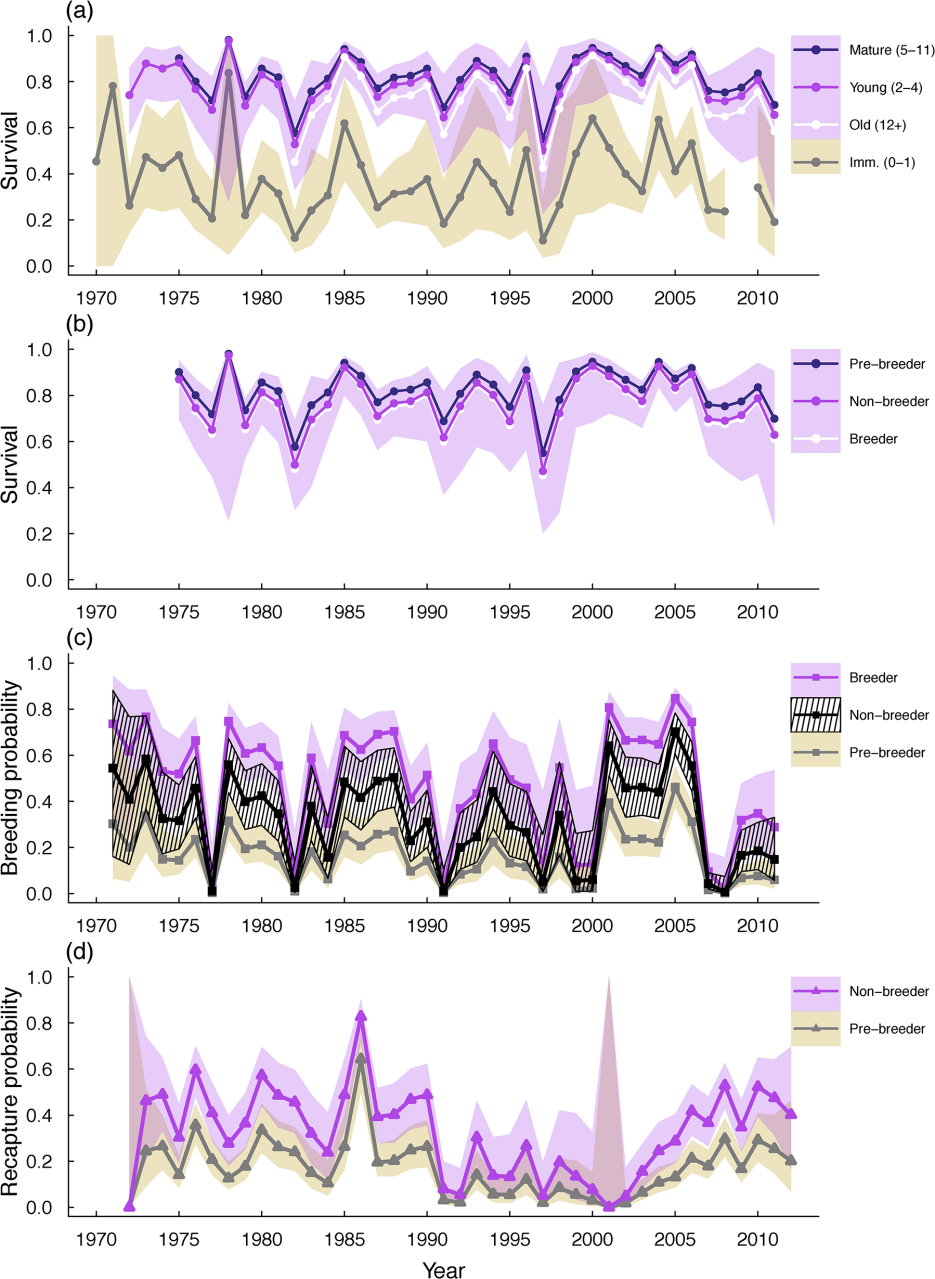
Time series of estimated (A, B) survival, (C) breeding probability, and (D) recapture probability from the reference model (*Ref*_*t*_). Survival estimates in (A) are for pre-breeders of all age classes. Shaded areas indicate 95% confidence intervals. For clarity, only the confidence intervals for young adult and immature pre-breeders are shown in (A). Survival differences for breeding states shown in (B) are for the mature age class (age 5–11). Confidence intervals for survival of different breeding states almost completely overlap so only the CI for non-breeders (middle line) is shown here. Recapture probabilities of breeders were estimated near one and are not shown in (D).

The reference model (*Ref*_*t*_) selected during the structural model selection included effects of age, breeding state, and year in all three parameters (S4 Table). The age structure selected differed for each parameter. For survival, age was best modeled as four classes (immature = age 0–1, young adult = age 2–4, mature adult = age 5–11, and old adult = age 12+; S4 Table). The survival of the oldest age class (age 12+) was lower than either young or mature adults, possibly indicating a senescent decline in survival after age 12. For recapture probability, models with either two or four age classes had similar support after the first step of structural model selection (ΔQAICc ~2). We carried both age structures forward through the next two steps at which point models with the two and four age classes still had nearly equal support (ΔQAICc < 2) so we proceeded with the more parsimonious structure (2 age classes, immature = age 0–1, and adult = age 2+). We also viewed this age structure as the most biologically realistic since it is likely most age related differences in recapture probability are driven by low colony visitation rates for immature individuals (age 0–1) compared to adults (age 2+) rather than differences among adults of different age categories [25]. The effect of age on breeding probability (transition into breeder state) was best modeled as an inverse relationship (1/age, S4 Table). Breeding state influenced both survival and breeding probability with survival of breeders lowest and pre-breeders highest, possibly indicating a cost of reproduction (Fig 2B). However, breeding probability in year t was highest for individuals that had bred in the previous year (t-1), suggesting once an individual starts to breed they are more likely to breed regardless of the potential cost.

Recapture probabilities for the first age class were very low (range = 0–0.01, Fig 2D) generating higher uncertainty around the survival estimates for immature individuals (Fig 2A). The recapture probability for individuals in the breeder state was near a boundary (near one) and the initial reference model was unable to resolve an estimate for this parameter. We re-ran the structural reference model using the simulated annealing algorithm in Program MARK. During numerical optimization in MARK, the simulated annealing algorithm periodically makes a random jump to a new parameter value to ensure that the optimization finds the global maximum rather than a local maximum [21]. The model results using simulated annealing were virtually identical, confirming that the estimates were from the global maximum. The estimate for breeder recapture probability improved slightly but most annual recapture values for the breeder state were still estimated near 1 (breeder range = 0.998–1, mean = 0.999). The high recapture probability for breeding individuals is likely a result of breeding site fidelity and the extended recapture period (~5 months). An individual breeding in the study area is likely to return the following year to breed in the same area [25] and would have many opportunities to be seen and resighted throughout the course of the breeding season. Recapture probabilities from 2000–2002 were exceptionally low (Fig 2D) and in one year (2001) were estimated to be essentially zero, consistent with a known reduction in resighting effort during that period. The low resighting probability resulted in higher uncertainty around survival and breeding probabilities from 1999–2002 and these estimates should be viewed with caution (Fig 2).

### Relationship with ocean covariates

**Fig 3:**
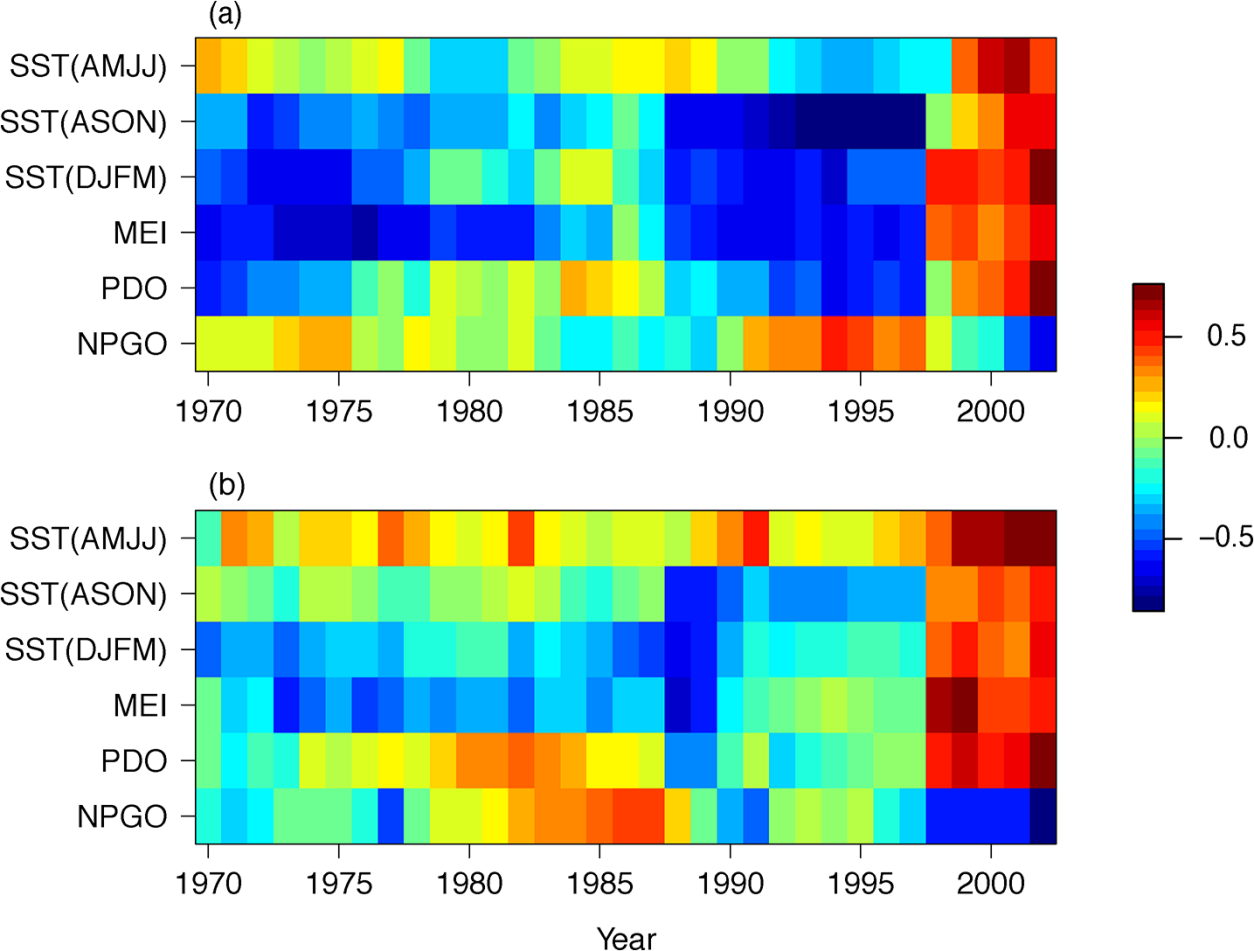
Sliding correlation between ocean covariates and Brandt’s cormorant (A) survival and (C) breeding probability. Year on the x-axis is the first year of the 10-year correlation window. The correlation varied with time but almost all variables showed a change in the correlation when 2007 entered the correlation window (i.e. in the 1998–2007 window). Correlations shown are for 6-year-old breeders.

For both survival and breeding probability, the models including the After07 interaction were strongly supported compared to models with no interaction (Tables 1 and 2), indicating that the relationship between oceanographic conditions and the demography of Brandt’s cormorant changed in the last five years. The quadratic relationship was favored over the linear model for all covariates (with the exception of NPGO for survival; Tables 1 and 2) and allowing the relationship with oceanographic covariates to be different after 2007 improved the R^2^ Dev of the quadratic model from 0.49 to 0.63 for the top covariate model of survival (Table 1), and from 0.22 to 0.50 for the top covariate model of breeding probability (Table 2). The sliding correlation analysis confirmed that the change in relationships between ocean covariates and both survival and breeding probability occurred between 2007/2008 (Fig 3). The correlation varied in time and appeared weak during some periods, in particular during the 1980s. This pattern may have at least partially occurred because the relationship was actually quadratic and not captured well by the linear correlation. Nonetheless, almost all variables showed a change in the sign of the correlation coefficient, generally shifting from negative to positive (with the exception of the NPGO which shifted to a negative correlation) when 2007 entered the correlation window (i.e. in the 1998–2007 window).

**Table 1:** Environmental covariate models for survival (S). Models were ranked and weighted within covariate categories by QAICc. Model structure: S (4 age class + state + **cov**) *p* (2 age class + state + time) Ψ (1/age + state + time).

S (4 age class + state + …)	k	QAICc	ΔQAICc	Weight	QDeviance	R^2^ Dev
MEI + MEI^2^ *After07	102	12959.93	0	1.00	3403.96	0.63
MEI + MEI^2^	99	12993.11	33.18	0	3443.22	0.49
MEI*After07	100	13044.43	84.50	0	3492.51	0.32
MEI	98	13068.92	108.99	0	3521.05	0.23
SST (ASON) + SST^2^ (ASON)*After07	102	12971.38	0	1.00	3415.41	0.59
SST (ASON)*After07	100	12983.71	12.33	0	3431.78	0.53
SST (ASON) + SST^2^ (ASON)	99	13001.43	30.05	0	3451.53	0.47
SST (ASON)	98	13016.87	45.49	0	3469.00	0.41
PDO + PDO^2^*After07	102	13070.00	0	0.75	3514.03	0.25
PDO*After07	100	13072.25	2.25	0.25	3520.33	0.23
PDO + PDO^2^	99	13090.70	20.70	0	3540.81	0.16
PDO	98	13099.75	29.75	0	3551.88	0.12
SST (DJFM) + SST^2^ (DJFM)*After07	102	13074.29	0	1.00	3518.32	0.24
SST (DJFM)*After07	100	13092.09	17.80	0	3540.16	0.16
SST (DJFM)+ SST^2^ (DJFM)	99	13096.32	22.03	0	3546.43	0.14
SST (DJFM)	98	13117.65	43.36	0	3569.78	0.06
NPGO *After07	100	13087.93	0	0.78	3536.01	0.18
NPGO + NPGO^2^ *After07	102	13090.45	2.52	0.22	3534.47	0.18
NPGO	98	13110.92	22.99	0	3563.05	0.08
NPGO + NPGO^2^	99	13112.82	24.89	0	3562.93	0.08
SST (AMJJ) + SST^2^ (AMJJ)*After07	102	13112.92	0	0.90	3556.95	0.10
SST (AMJJ)*After07	100	13117.38	4.46	0.10	3565.46	0.07
SST (AMJJ) + SST^2^ (AMJJ)	99	13122.43	9.51	0.01	3572.54	0.05
SST (AMJJ)	98	13126.80	13.88	0	3578.93	0.03

**Table 2:** Environmental covariate models for breeding probability (Ψ). Models were ranked and weighted within covariate categories by QAICc. Model structure: S (4 age class + state + time) *p* (2 age class + state + time) Ψ (1/age + state + **cov**).

Ψ (1/age + state + …)	K	QAICc	ΔQAICc	Weight	QDeviance	R^2^ Dev
SST (DJFM) + SST (DJFM)^2^ * After07	102	13075.56	0	1.00	3519.58	0.50
SST (DJFM) * After07	100	13121.47	45.91	0.00	3569.55	0.38
SST (DJFM) + SST (DJFM)^2^	99	13193.83	118.27	0.00	3643.93	0.22
SST (DJFM)	98	13288.43	212.87	0.00	3740.56	0.00
MEI + MEI^2^ * After07	102	13086.11	0	1.00	3530.13	0.47
MEI * After07	100	13128.95	42.84	0.00	3577.03	0.37
MEI + MEI^2^	99	13222.84	136.73	0.00	3672.94	0.15
MEI	98	13288.35	202.24	0.00	3740.48	0.00
SST (AMJJ) + SST (AMJJ)^2^ * After07	102	13104.97	0	1.00	3549.00	0.43
SST (AMJJ) * After07	100	13136.82	31.85	0.00	3584.90	0.35
SST (AMJJ) + SST (AMJJ)^2^	99	13201.23	96.26	0.00	3651.33	0.20
SST (AMJJ)	98	13238.08	133.11	0.00	3690.21	0.11
NPGO + NPGO^2^ * After07	102	13137.62	0	1.00	3581.64	0.36
NPGO * After07	100	13156.12	18.50	0.00	3604.20	0.31
NPGO + NPGO^2^	99	13259.97	122.35	0.00	3710.08	0.07
NPGO	98	13263.34	125.72	0.00	3715.47	0.06
SST (ASON) + SST (ASON)^2^ * After07	102	13139.64	0	0.98	3583.66	0.35
SST (ASON) * After07	100	13147.48	7.84	0.02	3595.56	0.33
SST (ASON)	98	13288.36	148.72	0.00	3740.50	0.00
SST (ASON) + SST (ASON)^2^	99	13290.24	150.60	0.00	3740.35	0.00
PDO + PDO^2^ * After07	102	13155.42	0	0.55	3599.45	0.32
PDO * After07	100	13155.82	0.40	0.45	3603.90	0.31
PDO + PDO^2^	99	13248.92	93.50	0.00	3699.03	0.09
PDO	98	13271.10	115.67	0.00	3723.23	0.04

**Fig 4:**
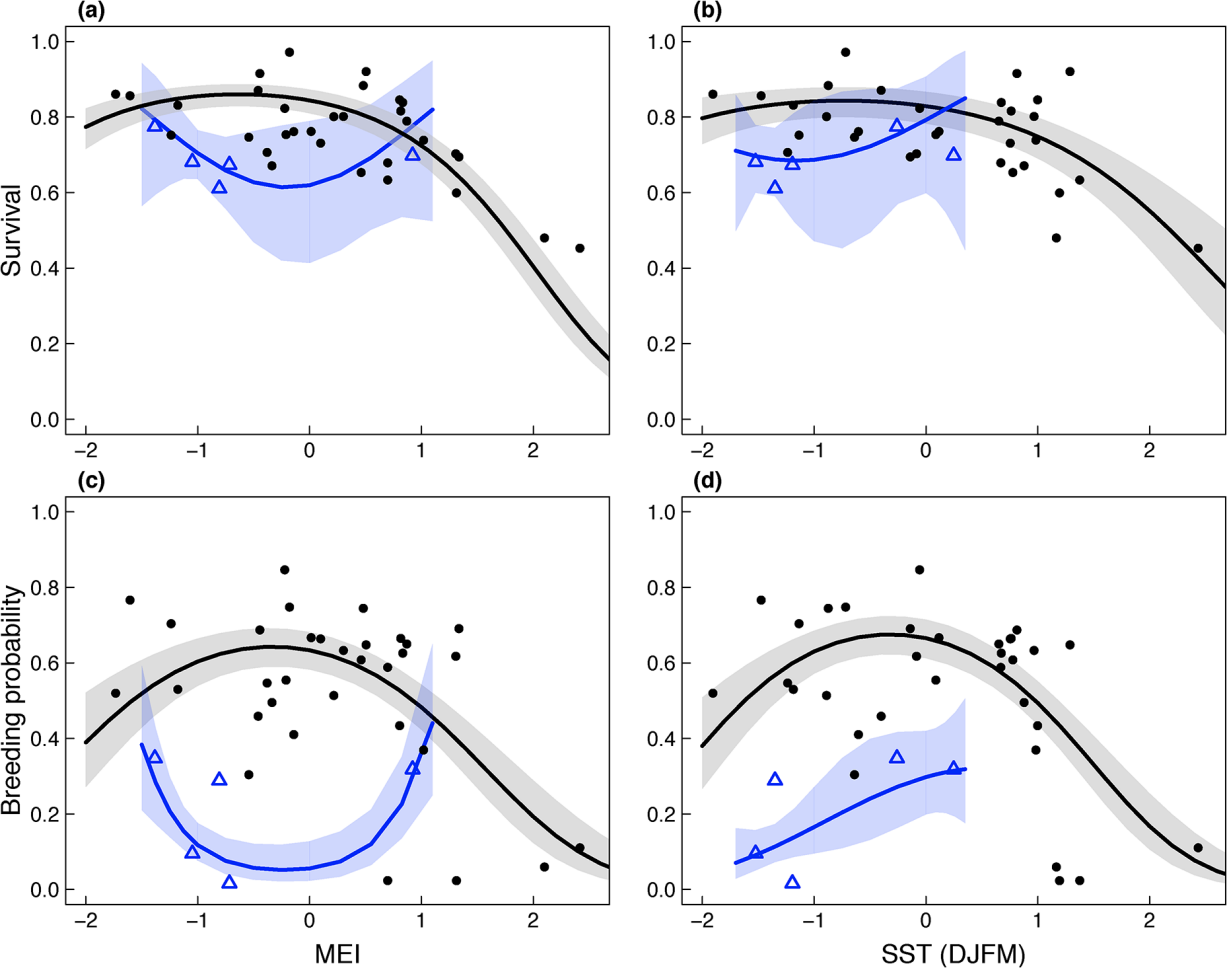
(A, B) Predicted relationships between survival, (C, D) breeding probability and important environmental covariates, the multivariate ENSO index (MEI), and winter (DJFM) sea surface temperature (SST). Points illustrate survival and breeding probability estimates from the reference model (*Ref*_*t*_) from 1972–2006 and open triangles are estimates from 2007–2011. For clarity, only relationships for age 6 breeders are shown. Relationships with other age classes and breeding states were similar due to the additive structure of the models. Estimates for the period when recapture probability was very low (2000–2002) are not shown.

The top covariate model for survival indicated a strong influence of El Niño variability on survival and included a quadratic relationship with MEI and the After07 interaction (R^2^ Dev = 0.63, F_(10, 36)_ = 6.10, *P* <0.001, Table 3 and S5 Table). The quadratic relationship between MEI and survival was effectively a threshold with moderate variability in survival at intermediate values of MEI and a strong decline in survival only with very strong El Niño events (positive values, Fig 4A). Local SST during the fall period (ASON) also accounted for a significant fraction of the temporal variability in survival (R^2^ Dev = 0.59, F_(10, 36)_ = 5.17, *P* <0.001, Table 3). No other covariate had significant support according to the ANODEV (Table 3).

**Table 3:** Top survival model for each covariate compared to reference (*Ref*_*t*_) and constant (*Ref*_*cst*_) models.

S (4 age class + state + …)	k	QAICc	ΔQAICc	Weight	QDeviance	R^2^ Dev	*P*-ANODEV
*Ref*_*t*_	138	12924.93	0.00	1	3295.80	1	NA
MEI + MEI^2^ *After07	102	12959.93	35.00	0	3403.96	0.63	0.000023
SST (ASON) + SST^2^ (ASON)*After07	102	12971.38	46.45	0	3415.41	0.59	0.00011
PDO + PDO^2^*After07	102	13070.00	145.07	0	3514.03	0.25	0.32
SST (DJFM) + SST^2^ (DJFM)*After07	102	13074.29	149.36	0	3518.32	0.24	0.38
NPGO *After07	100	13087.93	163.00	0	3536.01	0.18	0.44
SST (AMJJ) + SST^2^ (AMJJ)*After07	102	13112.92	187.99	0	3556.95	0.10	0.93
*Ref*_*cst*_	97	13132.95	208.02	0	3587.11	0	NA

In contrast, the top covariate model for breeding probability included a quadratic relationship with local wintertime SST (DJFM prior to breeding) and an After07 interaction. This model accounted for 50% of the temporal variability in breeding probability (R^2^ Dev = 0.50, F_10, 36)_ = 3.56, *P* = 0.002, Table 2 and S6 Table) and indicated that breeding probability peaked with cool ocean conditions (slightly below average) in winter and decreased with warm, El Niño, winters as well as with very cold winters (Fig 4C and 4D). The next highest ranked covariate, MEI (quadratic model with After07 interaction), also accounted for about half of the temporal variability in breeding probability (R^2^ Dev = 0.47, F_(10, 36)_ = 3.23, *P* = 0.004, Table 4) and spring SST (AMJJ) was also influential (R^2^ Dev = 0.43, F_(10, 36)_ = 2.72, *P* = 0.013, Table 4).

**Table 4:** Top Ψ models for each covariate compared to reference (*Ref*_*t*_) and constant (*Ref*_*cst*_) models.

Ψ (1/age + state + …)	k	QAICc	ΔQAICc	weight	QDev	R^2^ Dev	*P*-ANODEV
*Ref*_*t*_	138	12924.93	0.00	1	3295.80	1.00	NA
SST (DJFM) + SST (DJFM)^2^ * After07	102	13075.56	150.63	0	3519.58	0.50	0.002
MEI + MEI^2^ * After07	102	13086.11	161.17	0	3530.13	0.47	0.004
SST (AMJJ) + SST (AMJJ)^2^ * After07	102	13104.97	180.04	0	3549.00	0.43	0.013
NPGO + NPGO^2^ * After07	102	13137.62	212.69	0	3581.64	0.36	0.062
SST (ASON) + SST (ASON)^2^ * After07	102	13139.64	214.71	0	3583.66	0.35	0.068
PDO + PDO^2^ * After07	102	13155.42	230.49	0	3599.45	0.32	0.126
*Ref*_*cst*_	97	13286.42	361.49	0	3740.58	0.00	NA

## Discussion

As global climate changes, novel conditions may give rise to new environment-species relationships as well as no-analogue communities. Even though novel ocean conditions may already be apparent in the North Pacific and elsewhere, the vulnerability of environment-species relationships to change has not been widely addressed. Using over 40 years of demographic data, we found that the previously established relationships between ocean conditions and the demography of an upper trophic level marine predator have become unstable and unpredictable: beginning in 2007/2008, Brandt’s cormorant no longer responded to El Niño (MEI) and local sea surface temperature (SST) as they had for the previous three decades (Fig 4).

**Fig 5:**
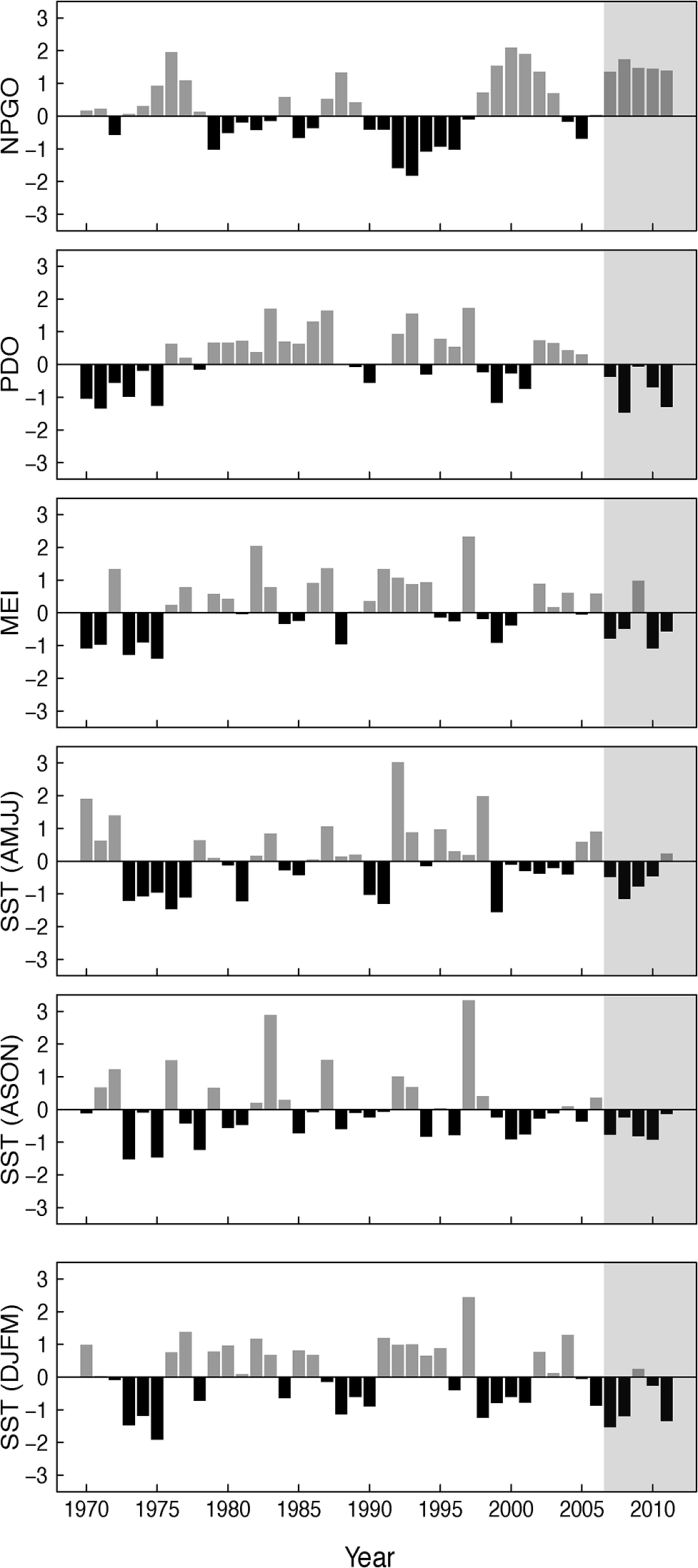
Standardized time series of ocean covariates. The annual indices NPGO, PDO, and MEI were averaged from April year t to March year t+1. Year on the x-axis refers to year t. Grey area covers the last five years when relationships changed, beginning with 2007.

This change coincided with a potential regime shift in broad-scale ocean conditions in the Northeast Pacific (Fig 5; [46]). The PDO, MEI, NPGO and local SST indices all indicate that cool conditions prevailed in four of the five years since 2006 (Fig 5). The shift in ocean conditions in 2007/2008 appeared to be similar in strength (though in the opposite direction) to the important regime shift from a cool to warm identified in 1976/1977 [46]. Although some ecological shifts were observed (e.g. seabird diet; [47]) the physical shift in 2007/2008 was not accompanied by the same broadly coherent ecological responses observed in 1976/1977 [46]. For Brandt’s cormorant, the return to cool conditions in 2007/2008 resulted in lower than expected survival and breeding probability. This result was especially noticeable for breeding probability: during the cool period of 2007–2011, the probability of breeding was so low that it was equivalent to what would have been predicted with a moderate to strong El Niño (Fig 4C and 4D).

The potential for relationships to change may be particularly likely for upper trophic level predators such as Brandt’s cormorant; for these species, the influence of the physical environment is often indirect, acting through changes in prey availability [48,49]. Because there may be one or more exchanges or trophic links between the physical environment and the response of an upper trophic level predator, there are more potential opportunities for altered species interactions to disrupt established links. Thus, the shifting relationship between ocean conditions and Brandt’s cormorant demography may be an indication of shifts in the food web and that the prey these birds depend on are no longer responding to cool, “productive,” ocean conditions in the same way. Indeed, evidence from seabird dietary studies suggests that a major change in forage fish community composition in central California occurred beginning in 2008, coinciding with the shift in the response of cormorants to ocean conditions [50].

Not only did the environment-demography relationships for Brandt’s cormorant change, they all changed at the same time, resulting in a simultaneous and unexpected decline in reproductive success [11], breeding probability, and survival (Fig 2). The decline over the last five years has serious implications for the population’s status. In several years virtually all cormorants skipped breeding (Fig 2C) and a year of reduced breeding probability typically coincided with a year of low survival (e.g. low survival from 1997–1998, coincided with low probability of transitioning into the breeding state between 1997–1998, resulting in reduced breeding in 1998; Fig 2). Combined with the low success of individuals that did breed [11], these recent changes almost certainly resulted in a substantial recruitment gap and a skewing of the age structure of the current breeding population towards older individuals, a situation that could significantly hamper population recovery.

Although the trends in survival and breeding probability were generally the same, there may be important differences in how these two parameters respond to ocean conditions. The responses of survival and breeding probability to ocean conditions were qualitatively similar, with both showing a quadratic response to most oceanographic variables. However, as expected, cormorant breeding probability was much more variable than survival (e.g. coefficient of variation of survival for age 6 breeder = 0.15, CV of breeding probability for age 6 breeders = 0.63, Fig 2) and more sensitive to environmental variation (i.e. steeper curve; Fig 4).

The model results also indicate that the environment may be acting on each demographic parameter in distinct ways. Brandt’s cormorants disperse away from the breeding colony in winter [51] thus their survival is dependent on ocean conditions integrated over relatively broad spatial and temporal scales. This was reflected in the model results as the basin-scale annual El Niño variable, MEI, had the strongest influence on survival. Although local SST influenced both survival and breeding probability, the timing differed, and may indicate there are critical periods that are unique to each demographic parameter. Fall SST was more influential for survival, perhaps reflecting the need for favorable post-breeding conditions to recover from the energetic cost associated with breeding. Whereas the strong influence of local winter SST on breeding probability may be more of a reflection of the importance of wintertime conditions in “priming” the California Current for summertime productivity [52]. The difference in timing may also have important implications for how the population responds to El Niño events: if the onset of an El Niño event is early enough to affect local SST in the fall, then it may have a greater impact on survival than if the El Niño arrives later in the winter when it would primarily impact breeding probability.

The results here indicated that the oldest age class had the lowest survival, perhaps pointing to a senescent decline in survival (Fig 2A). There was also a possible cost of reproduction detected, with breeders having the lowest survival and prebreeders the highest (Fig 2B). A potential cost of reproduction in this species was also noted by Nur and Sydeman [17]. The cost of reproduction in seabirds has been shown to vary with experience and individual quality [53–55] factors that were not included here. The fact that differences in survival between older individuals and breeding states were detected even without taking into account these important factors suggests that these may be important general patterns of survival in this population. However, further analysis investigating the effects of individual quality, breeding experience, and interactions between these factors and environmental conditions on survival and breeding probability is warranted.

The unusual response by Brandt’s cormorant to recent ocean conditions highlights a potential concern with current approaches to predicting the effects of environmental change: if environment-species relationship are not stable, predicting the responses of species or communities to novel climate conditions will be uncertain at best. The limited number of data points after 2007 restricts our ability to precisely determine the form and stability of these “new” relationships. However, these data provide evidence for our primary conclusion that the historic relationships may be unreliable for projecting into the future. The shifting response of the central California marine food web may be just one example of a potentially much larger issue: as climate change disrupts complex trophic interactions, and no-analog communities emerge, novel and unexpected responses to physical forcing may become more common. Although some published examples of shifting relationships exist [56,57], this question has not yet received enough attention to enable estimates of the frequency of occurrence or the processes driving non-stationarity in different systems. With the increasing emphasis on using global circulation models to project population responses to climate change, it is particularly important for ecologists to first examine the underlying relationships between vital rates and environmental variables for stationarity. Ultimately, developing more mechanistic, process-based models will be critical for understanding and accurately predicting the impact of climate change on upper trophic level species and communities.

## Supporting Information

S1 Fig Brandt’s cormorant in breeding plumage on the Farallon Islands. (JPG)

S2 Fig The number of chicks banded each year in the study colony on Southeast Farallon Island. (DOCX)

S1 Table Goodness-of-fit statistics for the global multistate test in U-CARE. (DOCX)

S2 Table Data sources and additional information for oceanographic covariates. (DOCX)

S3 Table Correlation between oceanographic variables used in this analysis. (DOCX)

S4 Table Steps of structural model selection. In step one and step two, models with two different age class structures had strong support and both of these were carried forward until step three. After step three, the more parsimonious and biologically realistic age class structure was carried forward.
(DOCX)

S5 Table All ocean covariate models for survival (S) ordered by QAICc and compared to year dependent reference model (*Ref*_*t*_) and constant (*Ref*_*cst*_) model. (DOCX)

S6 Table All ocean covariate models for breeding probability (Ψ) compared to year dependent reference model (*Ref*_*t*_) and constant (*Ref*_*cst*_) model. (DOCX)
