# Supplementary figures and images for: Shifting Effects of Ocean Conditions on Survival and Breeding Probability of a Long-Lived Seabird
Source: PLoS One. 2015 Jul 13;10(7):e0132372. doi: 10.1371/journal.pone.0132372 (PMC4500586; doi:10.1371/journal.pone.0132372)

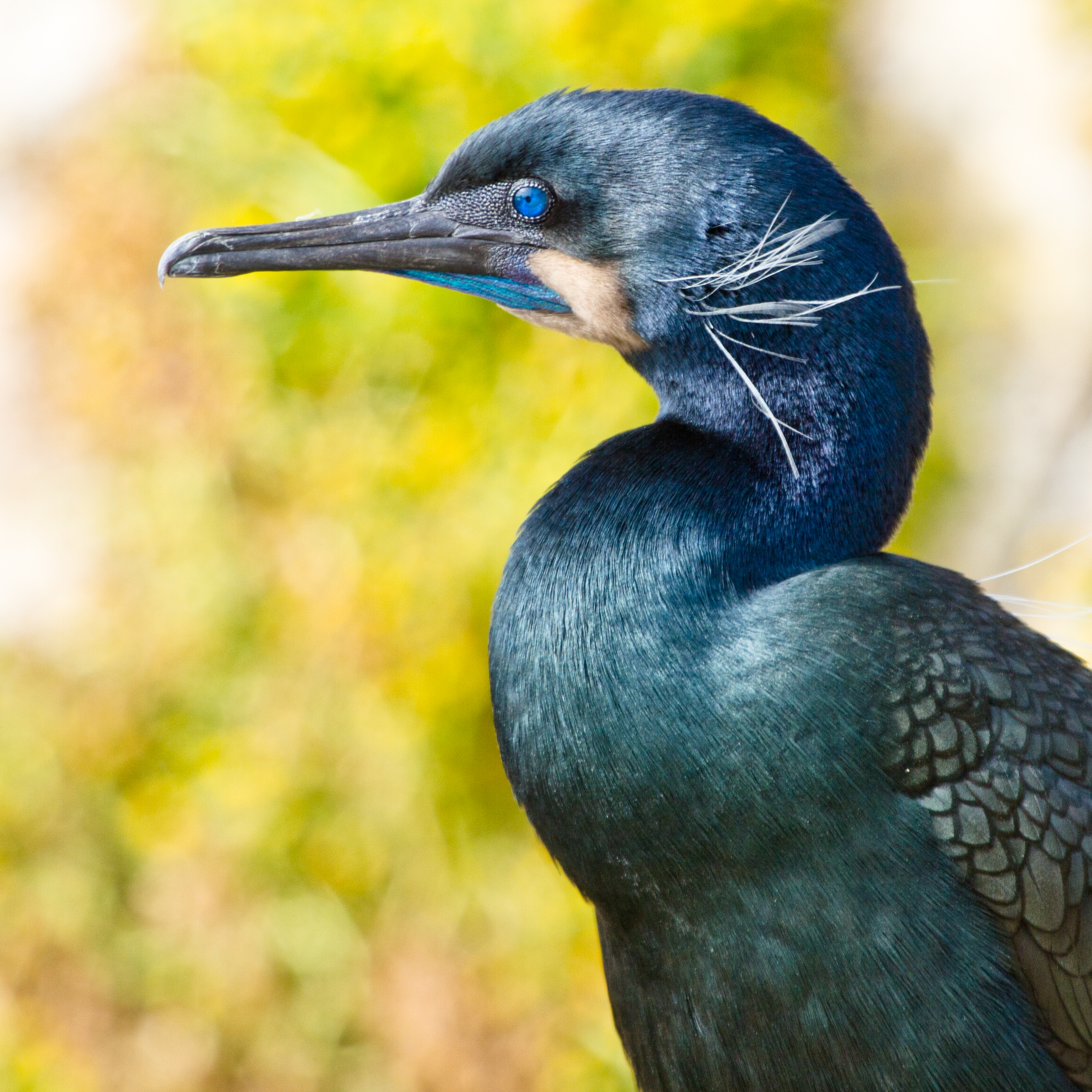

Supplement: S1 Fig — (JPG) [file pone.0132372.s001.jpg]
