# Supplementary material for: Shifting Effects of Ocean Conditions on Survival and Breeding Probability of a Long-Lived Seabird
Source: PLoS One. 2015 Jul 13;10(7):e0132372. doi: 10.1371/journal.pone.0132372 (PMC4500586; doi:10.1371/journal.pone.0132372)
Supplement: S2 Fig — (DOCX) [file pone.0132372.s002.docx]

**S2 Fig.** The number of chicks banded each year in the study colony on Southeast Farallon Island.
