# Supplementary material for: Shifting Effects of Ocean Conditions on Survival and Breeding Probability of a Long-Lived Seabird
Source: PLoS One. 2015 Jul 13;10(7):e0132372. doi: 10.1371/journal.pone.0132372 (PMC4500586; doi:10.1371/journal.pone.0132372)
Supplement: S1 Table — (DOCX) [file pone.0132372.s003.docx]

**S1 Table**. Goodness-of-fit statistics for the global multistate test in U-CARE.

| Test | Statistic | *P*-value | df |
| --- | --- | --- | --- |
| WBWA | 27.661 | 0.985 | 46 |
| 3G | 1880.649 | 0.000 | 251 |
| M | 250.774 | 0.000 | 56 |
| GOF test for JMV model | 2131.423 | 0.000 | 307 |
