# Supplementary material for: Shifting Effects of Ocean Conditions on Survival and Breeding Probability of a Long-Lived Seabird
Source: PLoS One. 2015 Jul 13;10(7):e0132372. doi: 10.1371/journal.pone.0132372 (PMC4500586; doi:10.1371/journal.pone.0132372)
Supplement: S2 Table — (DOCX) [file pone.0132372.s004.docx]

**S2 Table. Data sources and additional information for all oceanographic indices used.**

| Data | Time Scale | Source | URL | Processing |
| --- | --- | --- | --- | --- |
| Sea surface temperature (SST) | Daily | Point Blue Conservation Science, Scripps Institution of Oceanography Shore Station Program | <http://shorestation.ucsd.edu/active/index_active.html> | Calculated a seasonal means from daily values. If more than 15 days were missing in a month, we did not calculate mean for that month and instead interpolated between monthly values for that year (applied to 13 months total, <3% of the monthly values) |
| Pacific Decadal Oscillation (PDO) | Monthly | Nathan Mantua, JISAO, University of Washington | <http://jisao.washington.edu/pdo/PDO.latest> | Calculated annual mean from April-March |
| North Pacific Gyre Oscillation Index (NPGO) | Monthly | Emmanuele Di Lorenzo, Georgia Institute of Technology | <http://www.o3d.org/npgo/npgo.php> | Calculated annual mean from April-March |
| Multivariate ENSO index (MEI) | Bimonthly | NOAA Earth System Research Laboratory | <http://www.esrl.noaa.gov/psd/enso/mei/table.html> | Calculated annual mean from April-March using every other bimonthly value |
