# Supplementary material for: Shifting Effects of Ocean Conditions on Survival and Breeding Probability of a Long-Lived Seabird
Source: PLoS One. 2015 Jul 13;10(7):e0132372. doi: 10.1371/journal.pone.0132372 (PMC4500586; doi:10.1371/journal.pone.0132372)
Supplement: S3 Table — (DOCX) [file pone.0132372.s005.docx]

**S3 Table. Correlations between the oceanographic variables used in the analysis**. ****p* <0.001, ** 0.001< *p* < 0.01, * 0.01< *p* < 0.05.

|  | PDO | NPGO | MEI | SST (ASON) | SST (DJFM) |
| --- | --- | --- | --- | --- | --- |
| PDO |  |  |  |  |  |
| NPGO | -0.45** |  |  |  |  |
| MEI | 0.65*** | -0.39** |  |  |  |
| SST (ASON) | 0.55*** | -0.22 | 0.57*** |  |  |
| SST (DJFM) | 0.63*** | -0.45** | 0.70*** | 0.49** |  |
| SST (AMJJ) | 0.25 | -0.43** | 0.22 | 0.38* | 0.17 |
