# Supplementary material for: Shifting Effects of Ocean Conditions on Survival and Breeding Probability of a Long-Lived Seabird
Source: PLoS One. 2015 Jul 13;10(7):e0132372. doi: 10.1371/journal.pone.0132372 (PMC4500586; doi:10.1371/journal.pone.0132372)
Supplement: S4 Table — In step one and step two, models with two different age class structures had strong support and both of these were carried forward until step three. After step three, the more parsimonious and biologically realistic age class structure was carried forward. (DOCX) [file pone.0132372.s006.docx]

**S4 Table. Steps of structural model selection.** In step one and step two, models with two different age class structures had strong support and both of these were carried forward until step three. After step three, the more parsimonious and biologically realistic age class structure was carried forward.

| Step 1: *p* age class select | S | *p* | Ψ | k | QAICc | ΔQAICc | weight |
| --- | --- | --- | --- | --- | --- | --- | --- |
|  | age (12+) + state + time | 4 age class + state + time | age (12+) + state + time | 160 | 12947.77 | 0.00 | 0.72 |
|  |  | 2 age class + state + time |  | 158 | 12949.81 | 2.04 | 0.26 |
| *Global Model* |  | age (12+) + state + time |  | 169 | 12955.31 | 7.55 | 0.02 |
|  |  | 1/age + state + time |  | 158 | 13051.70 | 103.93 | 0.00 |
|  |  | ln(age) + state + time |  | 158 | 13171.26 | 223.50 | 0.00 |
|  |  | age + age^2^ + state + time |  | 159 | 13182.64 | 234.87 | 0.00 |
|  |  | age + state + time |  | 158 | 13221.58 | 273.81 | 0.00 |
| Step 2: *p* structure select | | | | | | | |
|  | age (12+) + state + time | 4 age class + state + time | age (12+) + state + time | 160 | 12947.77 | 0.00 | 0.74 |
|  |  | 2 age class + state + time |  | 158 | 12949.81 | 2.04 | 0.26 |
|  |  | 4 age class + state |  | 119 | 13177.63 | 229.86 | 0.00 |
|  |  | 2 age class + state |  | 117 | 13182.07 | 234.30 | 0.00 |
|  |  | 4 age class + time |  | 158 | 13375.32 | 427.55 | 0.00 |
|  |  | 2 age class + time |  | 156 | 13458.02 | 510.25 | 0.00 |
|  |  | 4 age class |  | 117 | 13655.08 | 707.31 | 0.00 |
|  |  | 2 age class |  | 115 | 13720.21 | 772.44 | 0.00 |
| Step 3: Ψ age class select | | | | | | | |
|  | age (12+) + state + time | 4 age class + state + time | 1/age + state + time | 149 | 12930.19 | 0.00 | 0.64 |
|  |  | 2 age class + state + time | 1/age + state + time | 147 | 12931.44 | 1.25 | 0.34 |
|  |  | 4 age class + state + time | 4 age class + state + time | 151 | 12937.42 | 7.23 | 0.02 |
|  |  | 2 age class + state + time | 4 age class + state + time | 149 | 12940.95 | 10.76 | 0.00 |
|  |  | 4 age class + state + time | 2 age class + state + time | 149 | 12941.07 | 10.88 | 0.00 |
|  |  | 4 age class + state + time | age (12+) + state + time | 160 | 12947.77 | 17.57 | 0.00 |
|  |  | 2 age class + state + time | 2 age class + state + time | 147 | 12948.43 | 18.23 | 0.00 |
|  |  | 2 age class + state + time | age (12+) + state + time | 158 | 12949.81 | 19.61 | 0.00 |
| Step 3 cont.: Ψ age class select | S | *p* | Ψ | k | QAICc | ΔQAICc | weight |
|  | age (12+) + state + time | 4 age class + state + time | ln(age) + state + time | 149 | 12966.89 | 36.70 | 0.00 |
|  |  | 4 age class + state + time | age + age^2^ + state + time | 150 | 12968.37 | 38.18 | 0.00 |
|  |  | 2 age class + state + time | age + age^2^ + state + time | 148 | 12968.52 | 38.33 | 0.00 |
|  |  | 2 age class + state + time | ln(age) + state + time | 147 | 12969.00 | 38.80 | 0.00 |
|  |  | 4 age class + state + time | age + state + time | 149 | 13002.71 | 72.51 | 0.00 |
|  |  | 2 age class + state + time | age + state + time | 147 | 13009.06 | 78.86 | 0.00 |
| Step 4: Ψ structure | | | | | | | |
|  | age (12+) + state + time | 2 age class + state + time | 1/age + state + time | 147 | 12931.44 | 0.00 | 1.00 |
|  |  |  | 1/age + time | 144 | 13108.34 | 176.90 | 0.00 |
|  |  |  | 1/age + state | 106 | 13291.25 | 359.81 | 0.00 |
|  |  |  | 1/age | 103 | 13494.91 | 563.47 | 0.00 |
| Step 5: S age class select | | | | | | | |
|  | 4 age class + state + time | 2 age class + state + time | 1/age + state + time | 138 | 12924.93 | 0.00 | 0.87 |
|  | 2 age class + state + time |  |  | 136 | 12930.18 | 5.25 | 0.06 |
|  | 1/age + state + time |  |  | 136 | 12931.41 | 6.48 | 0.03 |
|  | age (12+) + state + time |  |  | 147 | 12931.44 | 6.51 | 0.03 |
|  | age + age^2^ + state + time |  |  | 137 | 13008.80 | 83.86 | 0.00 |
|  | ln(age) + state + time |  |  | 136 | 13033.87 | 108.94 | 0.00 |
|  | age + state + time |  |  | 136 | 13188.83 | 263.90 | 0.00 |
| Step 6: S structure | | | | | | | |
|  | 4 age class + state + time | 2 age class + state + time | 1/age + state + time | 138 | 12924.93 | 0.00 | 0.96 |
|  | 4 age class + time |  |  | 136 | 12931.20 | 6.27 | 0.04 |
|  | 4 age class + Time + Time^2^ |  |  | 97 | 13105.37 | 180. | 0.00 |
|  | 4 age class + Time |  |  | 96 | 13123.54 | 198.60 | 0.00 |
|  | 4 age class + state |  |  | 97 | 13132.95 | 208.02 | 0.00 |
|  | 4 age class |  |  | 95 | 13136.06 | 211.13 | 0.00 |
