# Supplementary material for: Shifting Effects of Ocean Conditions on Survival and Breeding Probability of a Long-Lived Seabird
Source: PLoS One. 2015 Jul 13;10(7):e0132372. doi: 10.1371/journal.pone.0132372 (PMC4500586; doi:10.1371/journal.pone.0132372)
Supplement: S5 Table — (DOCX) [file pone.0132372.s007.docx]

**S5 Table**. All ocean covariate models for survival (S) ordered by QAICc and compared to year dependent reference model (*Ref_t_*) and constant (*Ref_cst_*) model.

| **S (4 age class + state + …)** | k | QAICc | ΔQAICc | Weight | QDeviance | R^2^Dev |
| --- | --- | --- | --- | --- | --- | --- |
| *Ref_t_* | 138 | 12924.93 | 0.00 | 1 | 3295.80 | 1.00 |
| MEI + MEI^2^ * After07 | 102 | 12959.93 | 35.00 | 0 | 3403.96 | 0.63 |
| SST (ASON) + SST (ASON)^2^ * After07 | 102 | 12971.38 | 46.45 | 0 | 3415.41 | 0.59 |
| SST (ASON) * After07 | 100 | 12983.70 | 58.77 | 0 | 3431.78 | 0.53 |
| MEI + MEI^2^ | 99 | 12993.11 | 68.18 | 0 | 3443.21 | 0.49 |
| SST (ASON) + SST (ASON)^2^ | 99 | 13001.43 | 76.49 | 0 | 3451.53 | 0.47 |
| SST (ASON) | 98 | 13016.87 | 91.93 | 0 | 3469.00 | 0.41 |
| MEI * After07 | 100 | 13044.43 | 119.50 | 0 | 3492.51 | 0.32 |
| MEI | 98 | 13068.92 | 143.99 | 0 | 3521.05 | 0.23 |
| PDO + PDO^2^ * After07 | 102 | 13070.00 | 145.07 | 0 | 3514.03 | 0.25 |
| PDO * After07 | 100 | 13072.25 | 147.32 | 0 | 3520.33 | 0.23 |
| SST (DJFM) + SST (DJFM)^2^ * After07 | 102 | 13074.29 | 149.36 | 0 | 3518.32 | 0.24 |
| NPGO * After07 | 100 | 13087.93 | 163.00 | 0 | 3536.01 | 0.18 |
| NPGO + NPGO^2^ * After07 | 102 | 13090.45 | 165.51 | 0 | 3534.47 | 0.18 |
| PDO + PDO^2^ | 99 | 13090.70 | 165.77 | 0 | 3540.81 | 0.16 |
| SST (DJFM) * After07 | 100 | 13092.09 | 167.15 | 0 | 3540.16 | 0.16 |
| SST (DJFM) + SST (DJFM)^2^ | 99 | 13096.32 | 171.39 | 0 | 3546.43 | 0.14 |
| PDO | 98 | 13099.75 | 174.81 | 0 | 3551.88 | 0.12 |
| NPGO | 98 | 13110.92 | 185.99 | 0 | 3563.05 | 0.08 |
| NPGO + NPGO^2^ | 99 | 13112.82 | 187.89 | 0 | 3562.93 | 0.08 |
| SST (AMJJ) + SST (AMJJ)^2^ * After07 | 102 | 13112.92 | 187.99 | 0 | 3556.95 | 0.10 |
| SST (AMJJ) * After07 | 100 | 13117.38 | 192.45 | 0 | 3565.46 | 0.07 |
| SST (DJFM) | 98 | 13117.65 | 192.72 | 0 | 3569.78 | 0.06 |
| SST (AMJJ) + SST (AMJJ)^2^ | 99 | 13122.43 | 197.50 | 0 | 3572.54 | 0.05 |
| SST (AMJJ) | 98 | 13126.80 | 201.87 | 0 | 3578.93 | 0.03 |
| *Ref_cst_* | 97 | 13132.95 | 208.02 | 0 | 3587.11 | 0.00 |
| ***…p* (2 age class + state + time)** **Ψ (1/age + state + time)** | | | | | | |
