# Supplementary material for: Shifting Effects of Ocean Conditions on Survival and Breeding Probability of a Long-Lived Seabird
Source: PLoS One. 2015 Jul 13;10(7):e0132372. doi: 10.1371/journal.pone.0132372 (PMC4500586; doi:10.1371/journal.pone.0132372)
Supplement: S6 Table — (DOCX) [file pone.0132372.s008.docx]

**S6 Table**. All ocean covariate models for breeding probability (Ψ) compared to year dependent reference model (*Ref_t_*) and constant (*Ref_cst_*) model.

| **Ψ (1/age + state + …)** | **k** | **QAICc** | **ΔQAICc** | **Weight** | **QDeviance** | R^2^**Dev** |
| --- | --- | --- | --- | --- | --- | --- |
| *Ref_t_* | 138 | 12924.93 | 0.00 | 1 | 3295.80 | 1.00 |
| SST (DJFM) + SST (DJFM)^2^ * After07 | 102 | 13075.56 | 150.63 | 0 | 3519.58 | 0.50 |
| MEI + MEI^2^ * After07 | 102 | 13086.11 | 161.17 | 0 | 3530.13 | 0.47 |
| SST (AMJJ) + SST (AMJJ)^2^ * After07 | 102 | 13104.97 | 180.04 | 0 | 3549.00 | 0.43 |
| SST (DJFM) * After07 | 100 | 13121.47 | 196.53 | 0 | 3569.55 | 0.38 |
| MEI * After07 | 100 | 13128.95 | 204.02 | 0 | 3577.03 | 0.37 |
| SST (AMJJ) * After07 | 100 | 13136.82 | 211.89 | 0 | 3584.90 | 0.35 |
| NPGO + NPGO^2^ * After07 | 102 | 13137.62 | 212.69 | 0 | 3581.64 | 0.36 |
| SST (ASON) + SST (ASON)^2^ * After07 | 102 | 13139.64 | 214.71 | 0 | 3583.66 | 0.35 |
| SST (ASON) * After07 | 100 | 13147.48 | 222.54 | 0 | 3595.56 | 0.33 |
| PDO + PDO^2^ * After07 | 102 | 13155.42 | 230.49 | 0 | 3599.45 | 0.32 |
| PDO * After07 | 100 | 13155.82 | 230.89 | 0 | 3603.90 | 0.31 |
| NPGO * After07 | 100 | 13156.12 | 231.19 | 0 | 3604.20 | 0.31 |
| SST (DJFM) + SST (DJFM)^2^ | 99 | 13193.83 | 268.89 | 0 | 3643.93 | 0.22 |
| SST (AMJJ) + SST (AMJJ)^2^ | 99 | 13201.23 | 276.30 | 0 | 3651.33 | 0.20 |
| MEI + MEI^2^ | 99 | 13222.84 | 297.90 | 0 | 3672.94 | 0.15 |
| SST (AMJJ) | 98 | 13238.08 | 313.15 | 0 | 3690.21 | 0.11 |
| PDO + PDO^2^ | 99 | 13248.92 | 323.99 | 0 | 3699.03 | 0.09 |
| NPGO + NPGO^2^ | 99 | 13259.97 | 335.04 | 0 | 3710.08 | 0.07 |
| NPGO | 98 | 13263.34 | 338.41 | 0 | 3715.47 | 0.06 |
| PDO | 98 | 13271.10 | 346.17 | 0 | 3723.23 | 0.04 |
| *Ref_cst_* | 97 | 13286.42 | 361.49 | 0 | 3740.58 | 0.00 |
| MEI | 98 | 13288.35 | 363.41 | 0 | 3740.48 | 0.00 |
| SST (ASON) | 98 | 13288.36 | 363.43 | 0 | 3740.50 | 0.00 |
| SST (DJFM) | 98 | 13288.43 | 363.49 | 0 | 3740.56 | 0.00 |
| SST (ASON) + SST (ASON)^2^ | 99 | 13290.24 | 365.31 | 0 | 3740.35 | 0.00 |
| ***…p* (2 age class + state + time)** **S (4 age class + state + time)** | | | | | | |
